# Numerical Analysis of Inertia Forces in the Connecting Rod and Their Impact on Stress Formation

**DOI:** 10.3390/ma18061385

**Published:** 2025-03-20

**Authors:** Andrzej Chmielowiec, Weronika Woś, Jan Czyżewski

**Affiliations:** 1 The Faculty of Mechanics and Technology, Rzeszow University of Technology, 37-450 Stalowa Wola, Poland; weronikawos@prz.edu.pl; 2 Doctoral School of the Rzeszow University of Technology, 35-959 Rzeszow, Poland; j.czyzewski@prz.edu.pl

**Keywords:** connecting rod, inertia force, FEA, FEM, numerical analysis

## Abstract

This paper presents a comprehensive model for the inertia force field acting on a moving connecting rod. The derived formulas enable the accurate calculation of resultant inertia forces and their distribution on individual components for finite element analysis (FEA). The method applies to symmetrical and complex-shaped connecting rods, addressing challenges in modeling forces for asymmetrical designs. This work advances the precision of stress and vibration modeling in connecting rods, crucial for tribology and reliability studies. By improving the understanding of wear and failure mechanisms in reciprocating systems, it supports design optimization. The article presents the application of the proposed computational methods using three materials typically used for connecting rod construction: 42CrMo4, aluminum 2618, and Ti6Al4V. The presented results demonstrate how the material selection influences the total inertia force and the resulting stresses within the material. The numerical results are presented based on simulations conducted for two connecting rods of different sizes, operating at extremely different rotational speeds. The conducted analyses show that in the examined cases, rotational speed is the key factor influencing inertia stresses. The implementation, based on Open Source tools, allows a numerical analysis of inertia forces and stresses, with all the methods and models available in an open repository.

## 1. Introduction

To effectively investigate the forces and stresses occurring in the connecting rod during operation, it is essential to consider the latest advancements in measurement technologies and numerical methods. The measurement and simulation of forces, stresses, and deformations in components such as the connecting rod require high precision to ensure that the results accurately reflect real-world operating conditions. Recent advances in sensor technology, such as piezoelectric and strain gauge sensors, have enabled more accurate measurements of forces and stresses in dynamic environments, providing valuable data for the development of improved simulation models. These measurements, when combined with finite element modeling (FEM) and other computational techniques, allow for a deeper understanding of the behavior of connecting rods under various loading conditions. Examples of such studies can be found in the works of Ou et al. [1], who applied advanced data analysis for the evaluation of diesel engine performance, Gutiérrez-León et al. [2], who developed fault-tolerant systems for engine sensors, and Manjhi and Kumar [3], who performed precise heat flux measurements in internal combustion engines. Moreover, innovations in multi-physics simulations that combine mechanics, thermodynamics, and fluid dynamics have opened new possibilities for comprehensive analyses, addressing not only the mechanical integrity of the connecting rod but also the effects of lubrication and thermal conditions on its performance, as demonstrated in the work of Vladov et al. [4]. These developments are directly relevant to the topic of this paper, as the accurate modeling of inertia forces and their impact on stress distribution within the connecting rod requires a deep understanding of both the theoretical and empirical aspects of the system’s behavior. These studies provide a solid foundation for further enhancing modeling and analysis methods for forces in crank–piston mechanisms.

The impact of gas forces and inertia forces on the distribution of oil pressure in the lubricating gap was described by Razavykia et al. [5]. The publication used precise formulas for the inertia force field, but the determination of the forces themselves was based on a very rough estimation of the weight distribution between the big end and the small end of the connecting rod. Nevertheless, the publication clearly demonstrated how precise modeling currently needed to be to obtain reliable simulation results that reflect the actual operating conditions of the crank–piston mechanism. The influence of lubrication on the operation of the connecting rod is a highly relevant topic. For instance, He et al. [6] utilizes the SVM machine learning method, which, based on critical input parameters, allows for the assessment of the quality of the connecting rod bearing’s performance. In particular, the applied method can take into account the distribution of forces and stresses, enabling the algorithm to appropriately consider the inertia forces present in the analyzed system. The quality analysis of connecting rod bearing lubrication depending on the loads occurring in the crank–piston mechanism is also the subject of research conducted by Mangeruga et al. [7] and Sun et al. [8]. According to the studies by Yin et al. [9], the distribution of inertia forces also has a significant impact on the tribological wear of the bearings mounted at the big end of the connecting rod. This is due to the fact that they operate under very high loads and in exceptionally unfavorable thermal conditions. On the other hand, Morris et al. [10] considered inertia forces as significant components affecting the oil film thickness in the development of a hydrodynamic lubrication model for the big end bearing.

The analysis of the causes of connecting rod failures is also a highly relevant issue. The primary cause of such events is usually the stresses occurring during the operation of this component. A thorough analysis of the reasons behind the failure involves the need to model the loads and stresses present during operation. Inertia force plays a significant role in this area. For instance, Chao [11] observed the destruction of a connecting rod due to fatigue wear caused by the action of periodically changing forces. Zhu et al. [12] presented a dynamic lubrication analysis of a connecting rod, which resulted in a stress distribution considering both proper lubrication and its absence. The presented results clearly showed that the correct oil film thickness, dependent on the distribution of forces, clearances, oil viscosity, and surface roughness, reflected the real operating conditions much more accurately. Pan and Zhang [13], on the other hand, considered the friction coefficient between connecting rod components, which they related, among other factors, to the load values occurring during operation. Through conducted simulations, they were able to improve the cross-sectional design and oil orifice, resulting in an improved connecting rod and big end bearing with a higher fatigue safety factor. Croccolo et al. [14] analyzed the impact of tightening screw friction on the stresses generated in the connecting rod, which, combined with stresses from gas forces and inertia forces, can lead to serious operational issues. Jiang et al. [15], on the other hand, used the Bayesian inference to study the influence of various factors on the deformation behavior of the connecting rod during operation.

A significant research area concerning connecting rods involves studies using the FEM (Finite Element Method). The main topics considered in this context include the analysis of stresses and deformations and their impact on the reliability and various tribological aspects of connecting rod operation. For instance, Francisco et al. [16] combined FEM with a genetic algorithm to achieve a connecting rod’s big-end design with improved tribological properties. On the other hand, Witek and Zelek [17] used the FEM to analyze a connecting rod that had failed due to fatigue wear. The analysis, which took into account gas forces and inertia forces, demonstrated that the crack initiation sites were located in areas with the highest stresses, significantly influenced by the torque of the connecting rod bolts. Lee et al. [18] studied the problem of connecting rod buckling and its effect on lightweight designs of the rod shank. Meanwhile, Seyedzavvar and Seyedzavvar [19] presented the results of applying the FEM in the design process of a connecting rod for a large marine engine. Shenoy and Fatemi [20] described the optimization of the connecting rod geometry for weight and cost reduction using the FEM, also analyzing material fatigue. Mekonen et al. [21] focused on analyzing the stress, deformation, fatigue strength, and lifespan of connecting rods in a diesel engine using four materials, with aluminum alloy providing the best results. On the other hand, Cheng et al. [22] analyzed the failure mechanism of a connecting rod, using dynamic simulations, finite element analysis, and fatigue damage theories to estimate its total and residual fatigue life, providing reliable data for future calculations. Ranjbarkohan et al. [23] analyzed the load and fatigue life of an engine’s connecting rod using Ansys (ver. 9) software to optimize durability and propose manufacturing improvements without the need for costly prototype testing. Muhammad et al. [24] reviewed the most important research on the design, the stress analysis and optimization of engine connecting rods using a finite element analysis in Ansys, emphasizing key parameters such as strain and fatigue to guide future automotive engineering work. Gopinath and Sushma [25] focused on the static stress analysis and mass optimization of connecting rods made of forged steel, aluminum, and titanium, and used the finite element analysis to investigate the potential for mass reduction under heavy cyclic load conditions in internal combustion engines. Zheng et al. [26] presented a 3D finite element analysis of an engine connecting rod, showing that the simulated stress distribution under maximum load conditions was consistent with the actual results and confirming the strength of the connecting rod.

From the point of view of an engine designer, knowing the forces acting on the connecting rod at a given crankshaft rotational speed is important, as it allows the determination of the allowable speed, so as to ensure safe engine operation. Connecting rod inertia forces were analyzed, among others, by Li [27], as well as Meng and Xiea [28]. The most advanced research works in this field were performed by Harrison [29] and Xie et al. [30], but even these studies could not provide a complete description of forces acting on the connecting rod. Harrison claimed that the proposed model was only a rough representation of actual conditions, based on the intuitive assumptions regarding the connecting rod’s behavior. Xie’s research mainly concerned an error analysis while using approximations for Taylor’s expansions. It should be noted that increasing the engine efficiency requirements in line with weight reduction cause the precise modeling of inertia forces on the connecting rod to be extremely important. This issue is very significant in terms of both connecting rod design and the analysis of vibration transferred to the crankshaft. The analytical and numerical methods described herein can be used for determining the field of forces for the purposes of stress analysis, a precise description of crankshaft vibrations, and the potential for damping these vibrations [31,32,33]. This would allow the further optimization of the connecting rod design, resulting in drive assembly weight reduction.

The primary goal of this paper was to develop formulas to enable the most accurate modeling of inertia forces generated in the crank and piston system.

## 2. Materials and Methods

**Figure 1:**
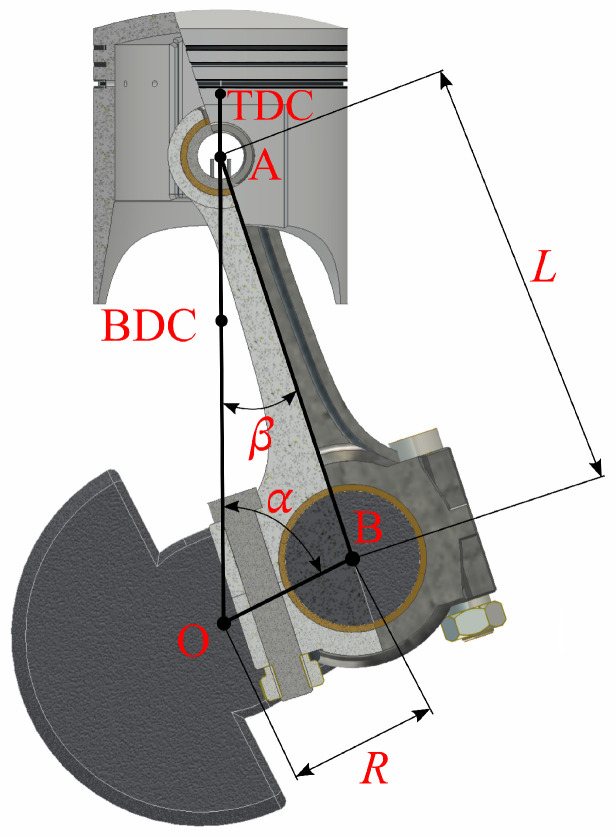
Scheme of the crank and piston system of a combustion engine.

Figure 1 shows a scheme of a typical crank–piston system, where the basic geometric quantities are marked. Later in the article, the notations below are used to describe the inertia forces acting on the connecting rod:
*O*point at which the crankshaft axis of rotation crosses perpendicularly the drawing plane;*A*point at which the connecting rod’s small-end axis of rotation crosses perpendicularly the drawing plane;*B*point at which the connecting rod’s big-end axis of rotation crosses perpendicularly the drawing plane;TDCtop dead center;BDCbottom dead center;*R*crank radius (equal to the distance between *O* and *B*);*L*distance between the axis of the connecting rod’s small-end hole and the axis of the big-end hole (equal to the distance between *A* and *B*);λconnecting rod’s length-to-crank’s length ratio, λ=R/L;αcrankshaft torsion angle;βconnecting rod’s torsion angle;ωcrankshaft angular speed;$m_{c}$connecting rod’s mass.

**Figure 2:**
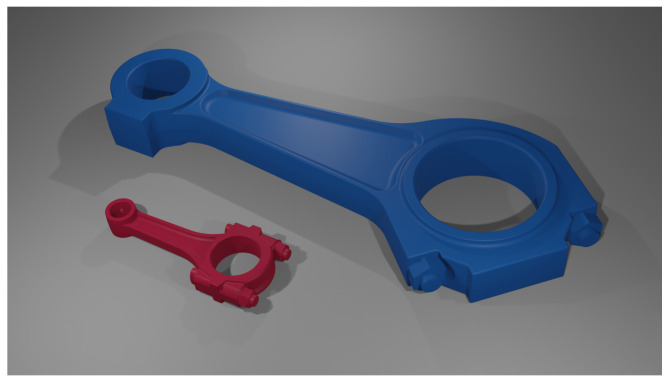
Connecting rods’ STL models for RÁBA-MAN D2356 HM6U (bigger) and Suzuki GS650 (smaller) engines.

Two connecting rods were selected for numerical simulations, the models of which are shown in Figure 2. The larger connecting rod came from the RÁBA-MAN D2356 HM6U (RÁBA, Győr, Hungary) 6-cylinder diesel engine, which was put into production in the late 1970s. The engine has a displacement of 10,690 cm^3^ and a power of 160.6 kW. The connecting rod centerline distance is 275 mm. This is a typical large diesel engine with a long crank radius, that achieves low operating speeds (maximum power at 2100 rpm). The cylinder bore is 121 mm, and the piston stroke is 150 mm. This engine was used, for example, in buses. The smaller connecting rod came from a Suzuki (Tokyo, Japan) GS650 4-cylinder gasoline motorcycle engine, which has a displacement of 650 cm^3^ and a power of 53.3 kW. It is a typical high-speed, short-stroke engine. The maximum power is reached at 9500 rpm. This engine has a cylinder diameter of 62 mm and a stroke of 55.8 mm. The distance between the axes of the connecting rod’s holes is 100 mm.

The key parameters of the RÁBA-MAN D2356 HM6U and Suzuki GS650 engines, along with information regarding the connecting rods, are presented in Table 1. These are used (later in the article) for modeling the inertia forces and the stresses they generate. The two described connecting rods were not selected randomly. The RABA-MAN connecting rod is an example of an asymmetric design, while the Suzuki one features full symmetry. Additionally, both connecting rods operate at completely different rotational speeds, which is a key factor influencing inertia.

### 2.1. Determination of Inertia Force by Weighing the Connecting Rod

As shown in [34], a typical approach to determine the inertia force acting on the connecting rod involves dividing the rod’s mass into two masses. The first mass, $m_{c,A}$, is concentrated at point A and generates an inertia force related to the reciprocating motion. The second mass, $m_{c,B}$, is concentrated at point B and undergoes rotational motion. The accelerations causing the inertia force at each point are defined by the following equations:

(1) $$\begin{array}{c} a_{A}=\left[\begin{array}{c} R\;\omega^{2}\left(\cos\alpha +\lambda \;\cos2\alpha \right)\\ 0\\ 0 \end{array}\right], \end{array}$$

(2) $$\begin{array}{c} a_{B}=\left[\begin{array}{c} R\omega^{2}\cos\alpha \\ -R\omega^{2}\sin\alpha \\ 0 \end{array}\right]. \end{array}$$

**Figure 3:**
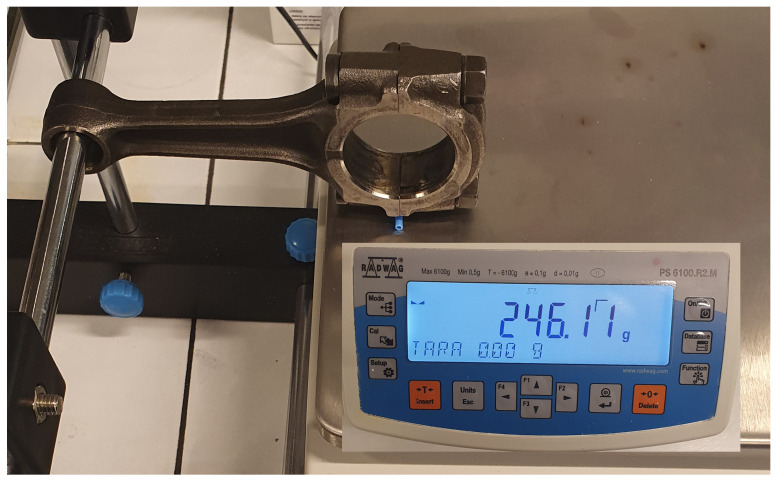
Weighing the connecting rod from a Suzuki GS650 engine, the weight indicates the fraction of the mass in rotation $m_{c,B}=0.24611\;\mathrm{kg}$.

The masses assigned to points A and B are determined during the process of weighing the connecting rod. The experimental determination of mass $m_{c,B}$ is shown in Figure 3, where the connecting rod for the Suzuki GS650 engine is being weighed.

The result obtained from weighing the connecting rod was almost identical to the numerical calculations. The relative error between the calculated value of 0.2468kg and the measured value of 0.24611kg was 0.3%. However, it should be noted that weighing requires a physical connecting rod and is not ideally suited to the design phase. This approach is also limited to symmetrical connecting rods (symmetrical to the plane passing through the axis of rotation of the connecting rod’s head and foot). For asymmetrical connecting rods, numerical solutions for the two- and three-dimensional cases should be applied.

### 2.2. Numerical Analysis Tools and Material Parameters

Mathematical packages, the Python (ver. 3.11) programming language with publicly available libraries, and the open-source programs Gmsh (ver. 4.13.1) [35] and FreeFem++ (ver. 4.15) [36] were used for numerical analysis. The STL models of connecting rods were selected as the starting point for the analysis. The STL is a  file format commonly used for 3D printing and computer-aided design (CAD). The name STL is an acronym that stands for stereolithography—a popular 3D printing technology. The format is also referred to as Standard Triangle Language or Standard Tessellation Language. Each file is made up of a series of linked triangles that describe the surface geometry of a 3D model or object. Mesh files with the appropriate level of precision were generated using Gmsh, followed by numerical integration and stress analysis using the FEM. The STL models, mesh files, and source codes are available in the open repository (accessed on 1 March 2025) https://github.com/achmie/conrod (accessed on 1 March 2025). The analysis covered three types of alloys commonly used in the production of connecting rods: steel 42CrMo4 [37], aluminum alloy 2618 [38], and titanium alloy Ti6Al4V [39,40]. Their physical properties are listed in Table 2. The introduced model assumed elastic material behavior within the simulated range. In the later sections of the article, it is explained why this approach is appropriate.

## 3. Theory and Calculations

In this part, three mathematical models of a connecting rod are introduced. The first is a one-dimensional model with a defined function of linear density. The second is a two-dimensional model with a defined function of planar density. The third is a three-dimensional model that most accurately describes inertia forces on the connecting rod. All three models were developed with the assumption that the connecting rod performed motion only in a plane perpendicular to the axis of crankshaft rotation. Considering the inertia forces acting on this component, this assumption is fully acceptable, as the inertia forces result from rotational motion around the shaft axis.

### 3.1. Equations of Motion

**Figure 4:**
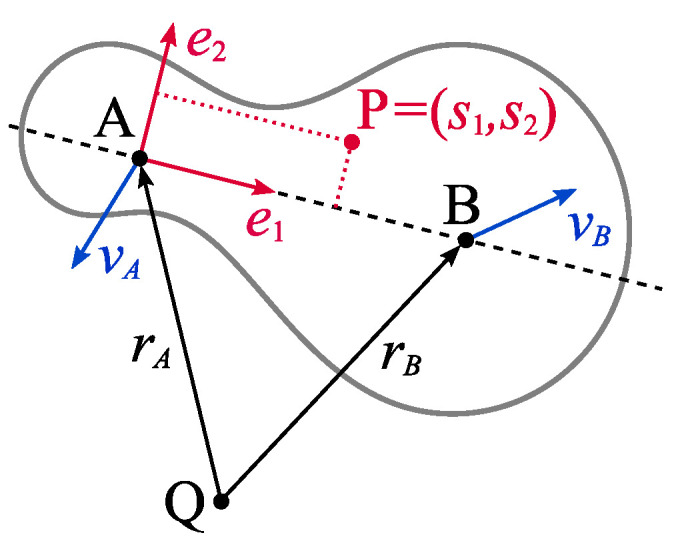
Rigid body in a two-dimensional space with two marked points *A* and *B* that define its motion.

It is assumed that the connecting rod is a rigid body that could move in two directions (Figure 4). Given that the distance between A and B is L, one can define versor $e_{1}=\frac{1}{L}\left(r_{B}-r_{A}\right)$. Since versor $e_{2}$ is obtained by rotating versor $e_{1}$ by angle $\frac{\pi }{2}$, the final form of these versors is as follows:

(3) $$\begin{array}{c} e_{1}=\frac{1}{L}\left(r_{B}-r_{A}\right), \end{array}$$

(4) $$\begin{array}{c} e_{2}=\frac{1}{L}\left[\begin{array}{cc} 0 & -1\\ 1 & 0 \end{array}\right]\left(r_{B}-r_{A}\right). \end{array}$$

If $P=\left(s_{1},s_{2}\right)=s_{1}e_{1}+s_{2}e_{2}$ is any point of the body in the coordinate system related to point *A*, then the position of that point in the coordinate system related to point *Q* can be described by the following equation:

(5) $$\begin{array}{c} r_{P}=\frac{1}{L}\left[\begin{array}{cc} L-s_{1} & s_{2}\\ -s_{2} & L-s_{1} \end{array}\right]r_{A}+\frac{1}{L}\left[\begin{array}{cc} s_{1} & -s_{2}\\ s_{2} & s_{1} \end{array}\right]r_{B}. \end{array}$$

Equation (5) clearly indicates that the position of point *P* is a linear function of the position of points *A* and *B*. The speed and acceleration equation at point *P*, correlated with the speed and acceleration of points *A* and *B* was obtained by time differentiation. It should be noted that Equation (5) is much simpler when the body is reduced to a rod placed on a line connecting points *A* and *B*. For these points, coordinate $s_{2}$ is 0 in all cases, while for $s=s_{1}$, the equation of position has the following form:

(6) $$\begin{array}{c} r_{P}=\frac{L-s}{L}r_{A}+\frac{s}{L}r_{B}. \end{array}$$

The equations of speed and acceleration are obtained simply by differentiation of the previous linear dependence. The double differentiation of relation (5) yields the formula for acceleration in the general case:   

(7) $$\begin{array}{c} a_{P}=\frac{1}{L}\left[\begin{array}{cc} L-s_{1} & s_{2}\\ -s_{2} & L-s_{1} \end{array}\right]a_{A}+\frac{1}{L}\left[\begin{array}{cc} s_{1} & -s_{2}\\ s_{2} & s_{1} \end{array}\right]a_{B}, \end{array}$$

and the double differentiation of relation (6) yields the formula:

(8) $$\begin{array}{c} a_{P}=\frac{L-s}{L}a_{A}+\frac{s}{L}a_{B}. \end{array}$$

Equation (5) allows us to describe the kinematics of the connecting rod and enables us to determine inertia forces on this part of the system. For further considerations, it is assumed that the origin of the coordinate system is point TDC. The *x* axis of the used coordinate system coincides with the line crossing points TDC and BDC. The *y* axis of the coordinate system is perpendicular to the *x* axis and crosses point TDC.

To describe the motion of the connecting rod, the relationships between angles α and β, shown in Figure 1, must be found. These quantities are related in the way defined by the following two equations:

(9) $$\begin{array}{c} \sin\beta =\frac{R}{L}\;\sin\alpha =\lambda \;\sin\alpha , \end{array}$$

(10) $$\begin{array}{c} \cos\beta =\sqrt{1-\lambda^{2}\sin^{2}\alpha }. \end{array}$$

**Figure 5:**
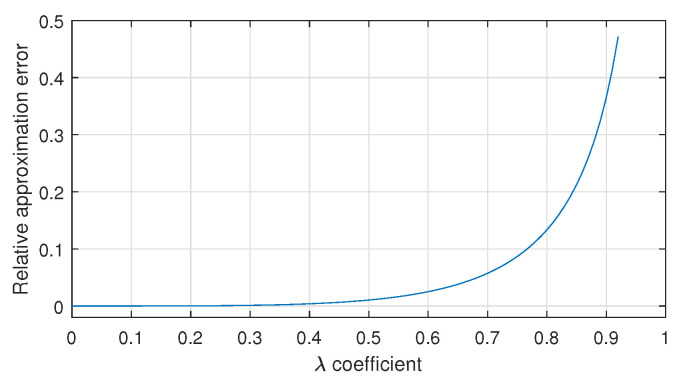
Relative error of the approximation $\sqrt{1-\lambda^{2}\sin^{2}\alpha }\approx 1-\frac{1}{2}\lambda^{2}\sin^{2}\alpha$ depending on the value of the coefficient λ.

To simplify (10), the Maclaurin series expansion of $\sqrt{1+x}$ is commonly used. The approximation $\cos\beta \approx 1-\frac{1}{2}\lambda^{2}\sin^{2}\alpha$ is very often used, regardless of the geometry of the crank–piston system. It turns out that for λ values not exceeding 0.5, the relative error of this approximation does not exceed 1%. However, for larger values of λ, there is a sharp increase in the error value, which is shown in Figure 5.

After further considerations, it is assumed that the function c(α) expresses the value of cosβ in the following way:

(11) $$\begin{array}{c} \cos\beta =1-\lambda \;c(\alpha ) \end{array}$$

and additionally, $c^{\prime }\left(\alpha \right)=\frac{dc}{d\alpha }$, $\dot{c}\left(\alpha \right)=\frac{dc}{dt}=\omega c^{\prime }\left(\alpha \right)$, $c^{\prime \prime }\left(\alpha \right)=\frac{d^{2}c}{d\alpha^{2}}$ and $\ddot{c}\left(\alpha \right)=\frac{d^{2}c}{dt^{2}}=\omega^{2}c^{\prime \prime }\left(\alpha \right)$. Each occurrence of c(α), $c^{\prime }\left(\alpha \right)$, and $c^{\prime \prime }\left(\alpha \right)$ can, for numerical calculations, be replaced by an exact formula:

(12) $$\begin{array}{c} c\left(\alpha \right)=\frac{1}{\lambda }\left(1-{\left(1-\lambda^{2}\sin^{2}\alpha \right)}^{1/2}\right), \end{array}$$

(13) $$\begin{array}{c} c^{\prime }\left(\alpha \right)=\frac{\lambda \sin2\alpha }{2{\left(1-\lambda^{2}\sin^{2}\alpha \right)}^{1/2}}, \end{array}$$

(14) $$\begin{array}{c} c^{\prime \prime }\left(\alpha \right)=\frac{\lambda \cos2\alpha }{{\left(1-\lambda^{2}\sin^{2}\alpha \right)}^{1/2}}+\frac{\lambda^{3}\sin^{2}2\alpha }{4{\left(1-\lambda^{2}\sin^{2}\alpha \right)}^{3/2}}, \end{array}$$

or approximated by several Maclaurin series terms:

(15) $$\begin{array}{c} c\left(\alpha \right)\approx \frac{1}{2}\lambda \sin^{2}\alpha -\sum_{n=2}^{N}{\left(-1\right)}^{n}\lambda^{2n-1}\frac{\sin^{2n}\alpha }{(2n)!}, \end{array}$$

(16) $$\begin{array}{c} c^{\prime }\left(\alpha \right)\approx \frac{1}{2}\lambda \sin2\alpha -\sum_{n=2}^{N}{\left(-1\right)}^{n}\lambda^{2n-1}\frac{\cos\alpha \;\sin^{2n-1}\alpha }{(2n-1)!}, \end{array}$$

(17) $$\begin{array}{c} c^{\prime \prime }\left(\alpha \right)\approx \lambda \cos2\alpha -\sum_{n=2}^{N}{\left(-1\right)}^{n}\lambda^{2n-1}\frac{2n\left(\cos^{2}\alpha +1\right)\sin^{2n-2}\alpha }{(2n-1)!}. \end{array}$$

As a result, the derived formulas allow for numerical computations with arbitrarily high precision.

It is now assumed that the vectors $r_{A}$ and $r_{B}$ are anchored at the TDC point, and their ends point to points A and B, respectively (Figure 1). The use of Formulas (9) and (11) gives the following dependencies

(18) $$\begin{array}{c} r_{A}=\left[\begin{array}{c} x_{A}\\ y_{A} \end{array}\right]=\left[\begin{array}{c} R\left(1-\cos\alpha +c(\alpha )\right)\\ 0 \end{array}\right]. \end{array}$$

(19) $$\begin{array}{c} r_{B}=\left[\begin{array}{c} x_{B}\\ y_{B} \end{array}\right]=\left[\begin{array}{c} R+L-R\cos\alpha \\ R\sin\alpha \end{array}\right]. \end{array}$$

When the position vectors of points *A* and *B* are known, it is possible to determine their speed and acceleration. This operation requires the time differentiation of $r_{A}$ and $r_{B}$. The differentiation is performed with the assumption that $\frac{d\alpha }{dt}=\omega =\mathrm{const}$. It must be emphasized that this approach is acceptable only if the inertia moment of the crankshaft with a flywheel is much higher than the inertia moment of the connecting rod attached to the piston. In other cases, a transfer of energy between all the parts of the crank and piston system must be considered. The assumption of constant angular speed ω allows us to describe the speed and acceleration of points *A* and *B* in the following way:

(20) $$\begin{array}{c} v_{A}=\frac{dr_{A}}{dt}=\frac{dr_{A}}{d\alpha }\cdot \frac{d\alpha }{dt}=\left[\begin{array}{c} R\;\omega \left(\sin\alpha +c^{\prime }\left(\alpha \right)\right)\\ 0 \end{array}\right], \end{array}$$

(21) $$\begin{array}{c} a_{A}=\frac{dv_{A}}{dt}=\frac{dv_{A}}{d\alpha }\cdot \frac{d\alpha }{dt}=\left[\begin{array}{c} R\;\omega^{2}\left(\cos\alpha +c^{\prime \prime }\left(\alpha \right)\right)\\ 0 \end{array}\right], \end{array}$$

(22) $$\begin{array}{c} v_{B}=\frac{dr_{B}}{dt}=\frac{dr_{B}}{d\alpha }\cdot \frac{d\alpha }{dt}=\left[\begin{array}{c} R\omega \sin\alpha \\ R\omega \cos\alpha \end{array}\right], \end{array}$$

(23) $$\begin{array}{c} a_{B}=\frac{dv_{B}}{dt}=\frac{dv_{B}}{d\alpha }\cdot \frac{d\alpha }{dt}=\left[\begin{array}{c} R\omega^{2}\cos\alpha \\ -R\omega^{2}\sin\alpha \end{array}\right]. \end{array}$$

### 3.2. The One-Dimensional Case

The first proposed model of connecting rod is one-dimensional. It reduces the conrod to a rod connecting its ends, crossing points *A* and *B*. It is also assumed that this rod has a certain linear density ρ(s). Let *C* be a point belonging to that rod. Its position is clearly determined by the distance from point *A*, i.e., $s\in [-l_{A},L+l_{B}]$, where $l_{A}$ is the distance of the edge of the connecting rod’s small end from point *A*, while $l_{B}$ is the distance of the edge of the connecting rod’s big end from point *B* (Figure 1). Referring to Figure 4 for the analyzed connecting rod, *Q* is the TDC, *P* is *C*, $s_{2}=0$, and $s_{1}=s$. Using Formula (6), one can determine the position of any point *C* as:

(24) $$\begin{array}{c} r_{C}=\frac{L-s}{L}r_{A}+\frac{s}{L}r_{B}, \end{array}$$

where $s\in [-l_{A},L+l_{B}]$. Using Equations (18) and (19), we obtain:

(25) $$\begin{array}{c} r_{C}=\left[\begin{array}{c} (L-s)\lambda \;c(\alpha )-R\cos\alpha +R+s\\ s\lambda \sin\alpha \end{array}\right], \end{array}$$

which, after double differentiation, allows us to obtain the following formula of acceleration at point *C*:

(26) $$\begin{array}{c} a_{C}=\frac{d^{2}r_{C}}{dt^{2}}=R\omega^{2}\left[\begin{array}{c} \frac{1}{L}\left(L-s\right)c^{\prime \prime }\left(\alpha \right)+\cos\alpha \\ -\frac{1}{L}s\sin\alpha \end{array}\right]. \end{array}$$

It is worth noting that Formula (26) is a generalization of Equations (21) and (23), obtained with the assumption of s=0 and s=L, respectively.

Performing integration with all the points of the rod, one can determine the resultant inertia force F on the connecting rod. In that situation, coordinates *x* and *y* of the inertia force can be described with the following formulas:

(27) $$\begin{array}{c} F_{x}=R\omega^{2}\left(\cos\alpha +c^{\prime \prime }\left(\alpha \right)\right)\;m_{c}-R\omega^{2}\frac{1}{L}c^{\prime \prime }\left(\alpha \right)\int_{-l_{A}}^{L+l_{B}}s\;\rho (s)\;ds, \end{array}$$

(28) $$\begin{array}{c} F_{y}=-R\omega^{2}\frac{1}{L}\sin\alpha \int_{-l_{A}}^{L+l_{B}}s\;\rho (s)\;ds. \end{array}$$

Assuming that

(29) $$\begin{array}{c} m_{c,0}=\int_{-l_{A}}^{L+l_{B}}s\;\rho (s)\;ds=\int_{-l_{A}}^{L+l_{B}}s\;dm \end{array}$$

is the first mass moment of length, and dividing the inertia force into a part related to the reciprocating motion of point *A* and a part related to the rotational motion of point *B*, *F* can be described as follows:

(30) $$\begin{array}{c} F=R\omega^{2}\left(\left(m_{c}-\frac{1}{L}m_{c,0}\right)\left[\begin{array}{c} \cos\alpha +c^{\prime \prime }\left(\alpha \right)\\ 0 \end{array}\right]+\frac{1}{L}m_{c,0}\;\left[\begin{array}{c} \cos\alpha \\ -\sin\alpha \end{array}\right]\right). \end{array}$$

The above derivation provides the basis for justifying the method of dividing the mass of the connecting rod into a part that remains in rotational motion and a part that remains in reciprocating motion. It should be emphasized that so far in publications [41,42], a different approach than presented in this article has prevailed. Namely, the mass of the connecting rod $m_{c}$ is divided into two parts: the rectilinear part $m_{c,A}$ and the rotating part $m_{c,B}$. Then, the mass $m_{c,A}$ is summed up with the mass of the piston, giving the total sum of the masses in the reciprocating motion $m_{l}=m_{p}+m_{c,A}$, and the mass $m_{c,B}$ is the total mass rotating. The division of the connecting rod mass into these two parts occurs in different proportions depending on the publication. The divisions found keep the proportions of the masses at 1:3 or 1:2 [41]. A division in the proportion *a*:*b* is often found in the literature, where *a* and *b* are lengths such that a+b=L, and the lengths of these sections depend on the position of the connecting rod’s center of gravity [43,44].

Generally, assume that $k_{1}$ is the rotating portion of the connecting rod mass. In this case, the inertia force generated by the masses in rotational motion is

(31) $$\begin{array}{c} F_{B}=k_{1}\;m_{c}\;a_{B}, \end{array}$$

where $k_{1}\in \left[0,1\right]$. Using Formula (23), we obtain:

(32) $$\begin{array}{c} F_{B}=k_{1}\;m_{c}\;R\omega^{2}\left[\begin{array}{c} \cos\alpha \\ -\sin\alpha \end{array}\right]. \end{array}$$

The motion of the remaining mass of the connecting rod generates the inertia force associated with the reciprocating movement, which can be expressed using Formula (21) as follows

(33) $$\begin{array}{c} F_{A}=\left(1-k_{1}\right)\;m_{c}\;a_{A}=\left(1-k_{1}\right)\;m_{c}\;R\omega^{2}\left[\begin{array}{c} \cos\alpha +c^{\prime \prime }\left(\alpha \right)\\ 0 \end{array}\right]. \end{array}$$

Comparing Equations (32) and (33) to Formula (30), it can be concluded that the value of $k_{1}$ should satisfy the equality

(34) $$\begin{array}{c} k_{1}\;m_{c}=\frac{1}{L}m_{c,0}=\frac{1}{L}\int_{-l_{A}}^{L+l_{B}}s\;\rho (s)\;ds. \end{array}$$

If one now examines the physical interpretation of the value $\frac{1}{L}m_{c,0}$, it turns out that it precisely represents the value indicated during the weighing process (Figure 3), when the support points of the connecting rod are the projections of points A and B along the axis perpendicular to the line AB.

### 3.3. The Two-Dimensional Case

The analysis of the one-dimensional case showed that the common practice of weighing the connecting rod’s big end has a deep theoretical justification. It turns out that this method is also correct for connecting rods for which the plane passing through the axis of rotation of the small end and the big end is also the plane of symmetry. However, with asymmetrical connecting rods, the situation is somewhat more complicated. It turns out that the big end’s weight approach may lead to errors in the determination of the resultant centrifugal force acting on the connecting rod.

**Figure 6:**
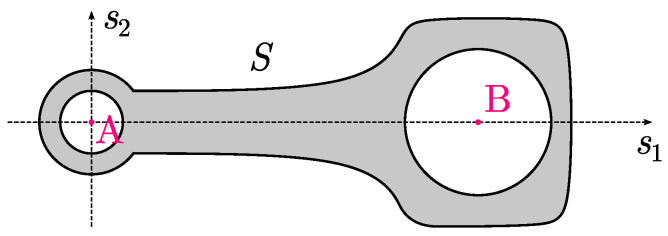
A flat model of the connecting rod with two points *A* (center of the small end) and *B* (center of the big end) that define its movement.

To take into account the asymmetry of the connecting rod, it was modeled by a certain flat surface *S* with a given surface density $\rho (s_{1},s_{2})$ as shown in Figure 6. The third dimension can be omitted assuming that there is no movement of the connecting rod towards the shaft axis. This assumption is generally valid as the energy of the connecting rod’s movement in this direction is negligibly small in relation to the energy of movement in the plane perpendicular to the shaft axis.

Taking into account Formulas (5), (18), and (19), one obtains the position vector of any point *P* on the surface

(35) $$\begin{array}{c} r_{P}=\left[\begin{array}{c} \left(L-s_{1}\right)\lambda \;c\left(\alpha \right)-R\cos\alpha -s_{2}\lambda \sin\alpha +s_{1}+R\\ -s_{2}\lambda \;c\left(\alpha \right)+s_{1}\lambda \sin\alpha +s_{2} \end{array}\right]. \end{array}$$

After double differentiating the above formula with respect to time, one obtains the formula for acceleration at the point *P*

(36) $$\begin{array}{c} a_{P}=R\omega^{2}\left[\begin{array}{c} c^{\prime \prime }\left(\alpha \right)-\frac{1}{L}s_{1}c^{\prime \prime }\left(\alpha \right)+\frac{1}{L}s_{2}\sin\alpha +\cos\alpha \\ -\frac{1}{L}s_{2}c^{\prime \prime }\left(\alpha \right)-\frac{1}{L}s_{1}\sin\alpha \end{array}\right]. \end{array}$$

Taking into account Formula (36), the components of the inertia force *F* are as follows

(37) $$\begin{array}{c} F_{x}=R\omega^{2}\left(\cos\alpha +c^{\prime \prime }\left(\alpha \right)\right)\;m_{c}+\\ R\omega^{2}\underset{S}{\;\int \int \;}\rho \left(s_{1},s_{2}\right)\left(\frac{1}{L}s_{2}\sin\alpha -\frac{1}{L}s_{1}c^{\prime \prime }\left(\alpha \right)\right)\;dS, \end{array}$$

(38) $$\begin{array}{c} F_{y}=R\omega^{2}\underset{S}{\;\int \int \;}\rho \left(s_{1},s_{2}\right)\left(-\frac{1}{L}s_{2}c^{\prime \prime }\left(\alpha \right)-\frac{1}{L}s_{1}\sin\alpha \right)\;dS. \end{array}$$

By introducing additional notations for the first moments in relation to the mass as below

(39) $$\begin{array}{c} m_{c,1}=\underset{S}{\;\int \int \;}s_{1}\;\rho \left(s_{1},s_{2}\right)\;dS=\underset{S}{\;\int \int \;}s_{1}\;dm, \end{array}$$

(40) $$\begin{array}{c} m_{c,2}=\underset{S}{\;\int \int \;}s_{2}\;\rho \left(s_{1},s_{2}\right)\;dS=\underset{S}{\;\int \int \;}s_{2}\;dm, \end{array}$$

it is possible to decompose the force of inertia into component vectors related to circular motion and reciprocating motion on the *x* and *y* axes

(41) $$\begin{array}{c} F=R\omega^{2}\left(\frac{1}{L}\;m_{c,1}\left[\begin{array}{c} \cos\alpha \\ -\sin\alpha \end{array}\right]+\left(m_{c}-\frac{1}{L}\;m_{c,1}\right)\left[\begin{array}{c} \cos\alpha +c^{\prime \prime }\left(\alpha \right)\\ 0 \end{array}\right]\right.\\ \;+\left.\frac{1}{L}\;m_{c,2}\left[\begin{array}{c} \sin\alpha \\ \cos\alpha \end{array}\right]-\frac{1}{L}\;m_{c,2}\left[\begin{array}{c} 0\\ \cos\alpha +c^{\prime \prime }\left(\alpha \right) \end{array}\right]\right). \end{array}$$

### 3.4. The Three-Dimensional Case

The three-dimensional case is directly obtainable from the two-dimensional formulas. This is due to the fact that the movement of the connecting rod along the third coordinate can generally be neglected. That is, the third coordinate of each of the connecting rod points can be considered a constant. Thus, the position and acceleration vectors in three dimensions take the form

(42) $$\begin{array}{c} r_{P}=\left[\begin{array}{c} \left(L-s_{1}\right)\lambda \;c\left(\alpha \right)-R\cos\alpha -s_{2}\lambda \sin\alpha +s_{1}+R\\ -s_{2}\lambda \;c\left(\alpha \right)+s_{1}\lambda \sin\alpha +s_{2}\\ s_{3} \end{array}\right], \end{array}$$

(43) $$\begin{array}{c} a_{P}=R\omega^{2}\left[\begin{array}{c} c^{\prime \prime }\left(\alpha \right)-\frac{1}{L}s_{1}c^{\prime \prime }\left(\alpha \right)+\frac{1}{L}s_{2}\sin\alpha +\cos\alpha \\ -\frac{1}{L}s_{2}c^{\prime \prime }\left(\alpha \right)-\frac{1}{L}s_{1}\sin\alpha \\ 0 \end{array}\right]. \end{array}$$

It is further assumed that the connecting rod is defined by a limited volume *V* with a specific density $\rho (s_{1},s_{2},s_{3})$. Thanks to these data, it is possible to express the value of auxiliary integrals

(44) $$\begin{array}{c} m_{c,1}=\underset{V}{\;\int \int \int \;}s_{1}\;\rho \left(s_{1},s_{2},s_{3}\right)\;dV=\underset{V}{\;\int \int \int \;}s_{1}\;dm, \end{array}$$

(45) $$\begin{array}{c} m_{c,2}=\underset{V}{\;\int \int \int \;}s_{2}\;\rho \left(s_{1},s_{2},s_{3}\right)\;dV=\underset{V}{\;\int \int \int \;}s_{2}\;dm. \end{array}$$

This leads to an analogous formula for the inertia force as for the two-dimensional case. The similarity of these formulas results directly from the assumption that the movement of the connecting rod only takes place in the plane perpendicular to the axis of rotation of the crankshaft, and therefore the third component of the inertia force must be zero.

(46) $$\begin{array}{c} F=R\omega^{2}\left(\frac{1}{L}\;m_{c,1}\left[\begin{array}{c} \cos\alpha \\ -\sin\alpha \\ 0 \end{array}\right]+\left(m_{c}-\frac{1}{L}\;m_{c,1}\right)\left[\begin{array}{c} \cos\alpha +c^{\prime \prime }\left(\alpha \right)\\ 0\\ 0 \end{array}\right]\right.\\ +\left.\frac{1}{L}\;m_{c,2}\left[\begin{array}{c} \sin\alpha \\ \cos\alpha \\ 0 \end{array}\right]-\frac{1}{L}\;m_{c,2}\left[\begin{array}{c} 0\\ \cos\alpha +c^{\prime \prime }\left(\alpha \right)\\ 0 \end{array}\right]\right). \end{array}$$

## 4. Results

To demonstrate the practical application of the introduced methods, a numerical study of the inertia forces acting on the connecting rod was conducted, and the FEM was used to analyze the stresses caused by these forces. As mentioned earlier, all calculations were performed using open-source software Python (ver. 3.11), Gmsh (ver. 4.13.1), FreeFem++ (ver. 4.15), and the source codes and materials necessary to replicate the study have been made available in the open repository https://github.com/achmie/conrod (accessed on 1 March 2025).

### 4.1. Numerical Determination of the Inertia Force Acting on the Connecting Rod

The formulas presented in the previous section were used in a numerical analysis of the connecting rod from the Suzuki GS650 engine, with the following parameters:

– Length of connecting rod L=0.1m;
– Crank radius R=0.0279m;
– Coefficient λ=0.279;
– Density of connecting rod material ρ=7800 kg/m^3^ for 42CrMo4, ρ=2760 kg/m^3^ for aluminum 2618 and ρ=4430 kg/m^3^ for Ti6Al4V;
– Shaft rotational speed f=9500rpm;
– Shaft angular speed $\omega =\frac{2\pi f}{60}=994.8\;\mathrm{rad}/s$.

In the case of this connecting rod, all the necessary calculations were performed using its MSH mesh, which was generated based on the STL model. It is worth noting that generating a mesh from the STL models can be problematic. Therefore, if possible, it is best to create the mesh based on various CAD formats. The Python code, which illustrates loading the STL model and numerically determining the volume *V* of the connecting rod, its mass $m_{c}$, and the moments $m_{c,1}$ and $m_{c,2}$, is provided in Appendix A.

**Figure 7:**
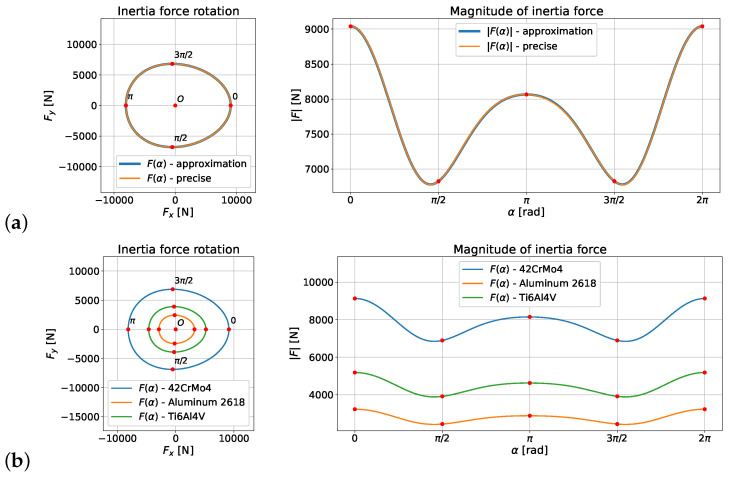
Curve determined by the inertia force vector F as a function of the α angle, defined by Equation (30) for the connecting rod of the Suzuki GS650 engine: (**a**) approximation and precise formulas for 42CrMo4; (**b**) precise formulas for 42CrMo4, aluminum 2618, and Ti6Al4V.

The calculated values formed the basis for the numerical determination of the inertia force using Equation (46). Figure 7 presents the shape of a curve determined by the inertia force vector F bound at the zero point of the coordinate system, and the diagram of length of vector F. The plot also shows that in the analyzed example, there was practically no difference between the application of the approximate Formula (15) and the precise Formula (12).

**Figure 8:**
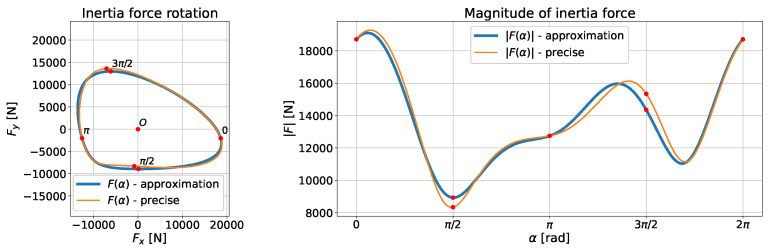
The curve defined by the vector of inertia force F as a function of the angle α given by Equation (41) for an exemplary connecting rod using both approximate Formula (15) and precise Formula (12).

Note that for symmetrical connecting rods, the value of $m_{c,2}$ is always equal to 0. In such cases, the inertia force looks analogous to the one-dimensional case. Interesting phenomena appear, however, for asymmetrical connecting rods, for which the value of $m_{c,2}$ is different from zero. Figure 8 shows such a situation. Those charts were generated for a connecting rod with the following parameters:

– Length of connecting rod L=0.2667m;
– Crank radius R=0.173m;
– Coefficient λ=0.6487;
– Weight of connecting rod $m_{c}=8.221\;\mathrm{kg}$;
– Shaft rotational speed f=1000rpm;
– Shaft angular speed ω=2πf/60=104.7rad/s;
– Part of connecting rod weight in rotational motion $\frac{1}{L}m_{c,1}=5.775\;\mathrm{kg}$;
– Part of the connecting rod mass unbalanced with respect to the axis connecting the center of the small end and the center of the big end $\frac{1}{L}m_{c,2}=1.6442\;\mathrm{kg}$.

Note that the connecting rod shown above is not real. The value of $\frac{1}{L}m_{c,2}$ has been intentionally overstated to make the chart readable. With real asymmetrical connecting rods, this weight is much lower. If one assumes that the $k_{2}\in \left[-1,1\right]$ parameter determines the degree of unbalance of the connecting rod relative to the $s_{2}$ axis, then $k_{2}\cdot m_{c}=\frac{1}{L}m_{c,2}$, and in this case, $k_{2}=0.2$. Figure 8 shows the curve defined by the inertia force vector F and the graph of the length of this vector as a function of the crankshaft torsion angle for the case where $k_{1}=0.7$ and $k_{2}=0.2$. There is an evident asymmetry in both graphs due to the fact that $m_{c,2}$ is nonzero. The plot also presents the difference in application of approximate Formula (15) and precise Formula (12).

### 4.2. Using FEM to Analyze Inertial Stresses

In the case of a homogeneous isotropic material, the state of static equilibrium between the displacement of the body u and the body force f causing it can be described by the following equation [45,46]:

(47) $$\begin{array}{c} \left(\lambda +\mu \right)\nabla \left(\nabla \cdot u\right)+\mu \nabla^{2}u+f=0, \end{array}$$

where $\lambda =\frac{E\nu }{\left(1+\nu )(1-2\nu \right)}$ and $\mu =\frac{E}{2(1+\nu )}$ are the Lame’s parameters, *E* is the Young’s modulus of the material, and ν is its Poisson’s ratio. Equation (47) is a direct consequence of the system of equations

(48) $$\begin{array}{c} \nabla \cdot \sigma +f=0, \end{array}$$

(49) $$\begin{array}{c} \sigma =C:\varepsilon , \end{array}$$

(50) $$\begin{array}{c} \varepsilon =\nabla^{s}u=\frac{1}{2}\left(\nabla u+\nabla u^{T}\right), \end{array}$$

where $\sigma =\sigma_{ij}$ is the stress tensor, $\varepsilon =\varepsilon_{ij}$ is the elementary strain tensor, $C=C_{ijkl}$ is the stiffness tensor, and the symbol : denotes the dot product of the second-order tensors. Using the assumption of isotropic material, one can define the stiffness tensor coefficients as $C_{ijkl}=\lambda \delta_{ij}\delta_{kl}+\mu \left(\delta_{ik}\delta_{jl}+\delta_{il}\delta_{jk}\right)$, where δ is the Kronecker delta.

One can reduce the problem thus posed to its weak formulation. Multiplying Equation (48) by the test function v and integrating using the Gauss–Green theorem, one obtains the formula [45]:

(51) $$\begin{array}{c} \int_{\Omega }\left(C:\varepsilon (u)\right):\varepsilon \left(v\right)\;d\Omega -\int_{\Omega }f\cdot v\;d\Omega -\int_{\partial \Omega }g\cdot v\;d(\partial \Omega )=0, \end{array}$$

where Ω is the domain, ∂Ω is the boundary of the domain, and g is the stress function imposed on the boundary of the domain. Finding function u, which for each test function v satisfies the above equation, is in fact a search for such a function u, which minimizes the energy of the system. In the case of the problem considered here, the relation g=0 also holds, because the behavior of the connecting rod is only analyzed under the influence of the inertial force field. Therefore, the weak formulation finally takes the form:

(52) $$\begin{array}{c} \int_{\Omega }\left(C:\varepsilon (u)\right):\varepsilon \left(v\right)\;d\Omega -\int_{\Omega }f\cdot v\;d\Omega =0, \end{array}$$

where body force f is determined by material density ρ(x,y,z) and inertia acceleration (43) in the following formula:   

(53) $$\begin{array}{c} f\left(x,y,z\right)=\rho \left(x,y,z\right)R\omega^{2}\left[\begin{array}{c} c^{\prime \prime }\left(\alpha \right)-\frac{x}{L}c^{\prime \prime }\left(\alpha \right)+\frac{y}{L}\sin\alpha +\cos\alpha \\ -\frac{y}{L}c^{\prime \prime }\left(\alpha \right)-\frac{x}{L}\sin\alpha \\ 0 \end{array}\right]. \end{array}$$

To find the function u in FreeFem++, it is necessary to write the integral expressions as arithmetic relations between functions and their derivatives.

For this purpose, the elementary deformation tensor is determined:

(54) $$\begin{array}{c} \begin{array}{cc} \varepsilon (u) & =\left[\begin{array}{ccc} \varepsilon_{xx}\left(u\right) & \varepsilon_{xy}\left(u\right) & \varepsilon_{xz}\left(u\right)\\ \varepsilon_{yx}\left(u\right) & \varepsilon_{yy}\left(u\right) & \varepsilon_{yz}\left(u\right)\\ \varepsilon_{zx}\left(u\right) & \varepsilon_{zy}\left(u\right) & \varepsilon_{zz}\left(u\right) \end{array}\right]\\ & =\left[\begin{array}{ccc} \frac{\partial u_{x}}{\partial x} & \frac{1}{2}\left(\frac{\partial u_{x}}{\partial y}+\frac{\partial u_{y}}{\partial x}\right) & \frac{1}{2}\left(\frac{\partial u_{x}}{\partial z}+\frac{\partial u_{z}}{\partial x}\right)\\ \frac{1}{2}\left(\frac{\partial u_{y}}{\partial x}+\frac{\partial u_{x}}{\partial y}\right) & \frac{\partial u_{y}}{\partial y} & \frac{1}{2}\left(\frac{\partial u_{y}}{\partial z}+\frac{\partial u_{z}}{\partial y}\right)\\ \frac{1}{2}\left(\frac{\partial u_{z}}{\partial x}+\frac{\partial u_{x}}{\partial z}\right) & \frac{1}{2}\left(\frac{\partial u_{z}}{\partial y}+\frac{\partial u_{y}}{\partial z}\right) & \frac{\partial u_{z}}{\partial z} \end{array}\right]. \end{array} \end{array}$$

Considering the fact that the stiffness tensor C for a homogeneous isotropic material can be written in the form of a matrix as

(55) $$\begin{array}{c} C=\left[\begin{array}{ccccccccc} \lambda +2\mu & \cdot & \cdot & \cdot & \mu & \cdot & \cdot & \cdot & \mu \\ \cdot & \lambda & \cdot & \mu & \cdot & \cdot & \cdot & \cdot & \cdot \\ \cdot & \cdot & \lambda & \cdot & \cdot & \cdot & \mu & \cdot & \cdot \\ ine\cdot & \mu & \cdot & \lambda & \cdot & \cdot & \cdot & \cdot & \cdot \\ \mu & \cdot & \cdot & \cdot & \lambda +2\mu & \cdot & \cdot & \cdot & \mu \\ \cdot & \cdot & \cdot & \cdot & \cdot & \lambda & \cdot & \mu & \cdot \\ ine\cdot & \cdot & \mu & \cdot & \cdot & \cdot & \lambda & \cdot & \cdot \\ \cdot & \cdot & \cdot & \cdot & \cdot & \mu & \cdot & \lambda & \cdot \\ \mu & \cdot & \cdot & \cdot & \mu & \cdot & \cdot & \cdot & \lambda +2\mu \end{array}\right], \end{array}$$

where · is equivalent to zero, the following relation holds

(56) $$\begin{array}{c} \left(C:\varepsilon (u)\right):\varepsilon \left(v\right)=\lambda \left(\nabla \cdot u\right)\left(\nabla \cdot v\right)+2\mu \left[\varepsilon \left(u\right):\varepsilon \left(v\right)\right]. \end{array}$$

If, in addition, the tensors ε(u) and ε(v) are treated as vectors of dimension nine, then the product ε(u):ε(v) gives the same value as the dot product of the vectors corresponding to these tensors. The fragment of code in FreeFem++ that defines the problem (56) is provided in Appendix B.

After determining the function u, it is possible to calculate the elementary strain tensor ε and the stress tensor σ. The obtained results were illustrated using the example of the von Mises stress $\sigma_{v}$, which averages all the elements of the stress tensor using the formula:

(57) $$\begin{array}{c} \sigma_{v}=\sqrt{\frac{1}{2}\left[{\left(\sigma_{xx}-\sigma_{yy}\right)}^{2}+{\left(\sigma_{yy}-\sigma_{zz}\right)}^{2}+{\left(\sigma_{zz}-\sigma_{xx}\right)}^{2}\right]+3\left(\sigma_{xy}^{2}+\sigma_{yz}^{2}+\sigma_{zx}^{2}\right)}. \end{array}$$

We emphasize that in this analysis, an elastic model was assumed throughout the entire simulation. The validity of this assumption was confirmed by the numerical results for the maximum von Mises stress values. The numerical convergence of the proposed model, depending on the mesh size, is presented in Table 3. The data were developed for a model operating with material properties typical for steel 42CrMo4 and an angle of α=0°. The mesh sizes analyzed were determined by scaling the dimensions used by Gao et al. [37]. It should be noted that the obtained maximum stress values were much lower than those reported in other publications [37,47,48,49]. This is because the analysis only considered inertia forces, without accounting for the gas forces. Including gas forces significantly alters both the distribution and magnitude of the stresses.

**Figure 9:**
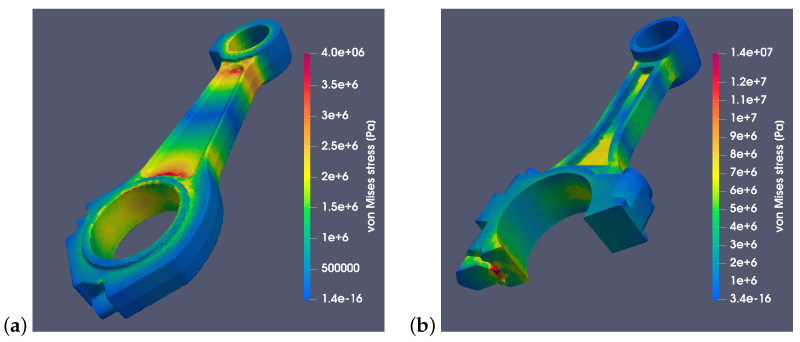
The highest values of von Mises stresses for: (**a**) RÁBA-MAN D2356 HM6U connecting rod made of steel 42CrMo4 at angle α=0°, (**b**) Cross-section of the connecting rod Suzuki GS650 made of steel 42CrMo4 at angle α=90°.

**Figure 10:**
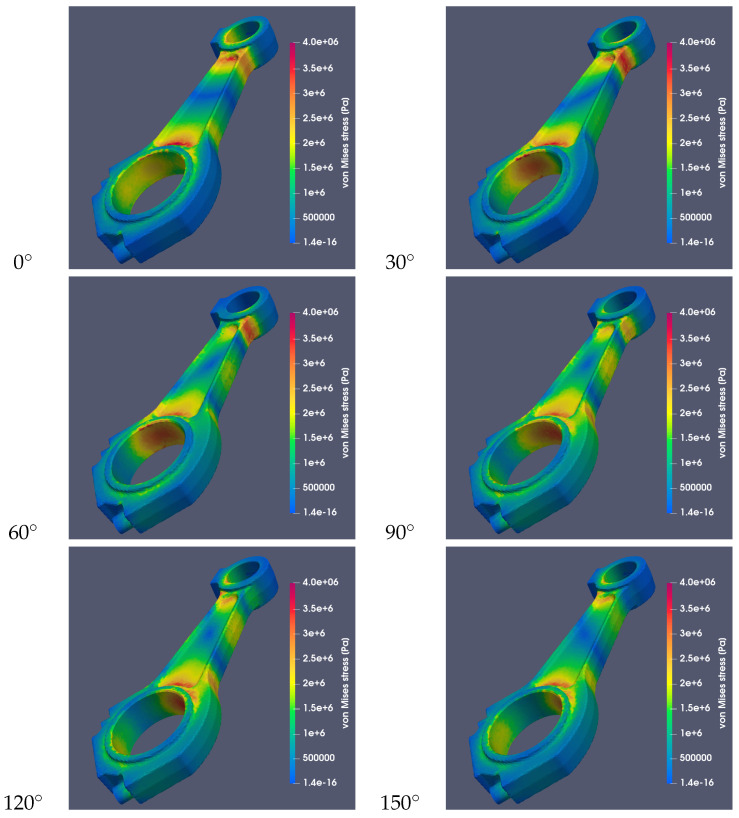
Von Mises stresses for a RÁBA-MAN D2356 HM6U connecting rod made of steel (St) at different values of the α angle with a common stress scale.

**Figure 11:**
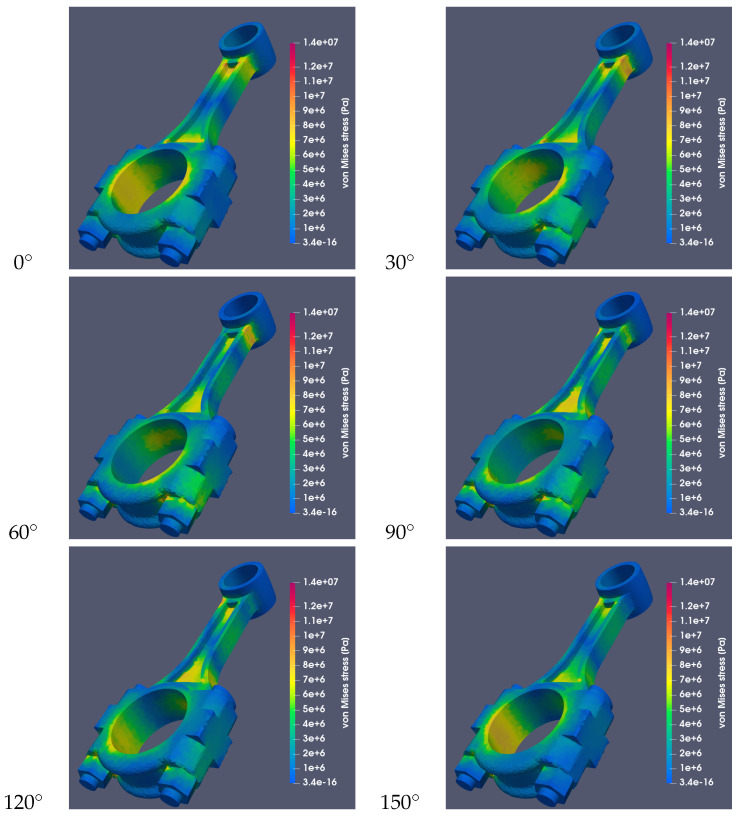
Von Mises stresses for a Suzuki GS650 connecting rod made of 42CrMo4 at different values of the α angle with a common stress scale.

**Figure 12:**
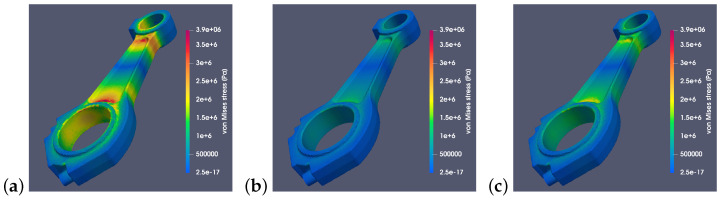
Von Mises stresses for a RÁBA-MAN D2356 HM6U connecting rod made of (**a**) steel 42CrMo4, (**b**) aluminum alloy 2618, and (**c**) titanium alloy Ti6Al4V with a common stress scale (for angle α=0°).

**Figure 13:**
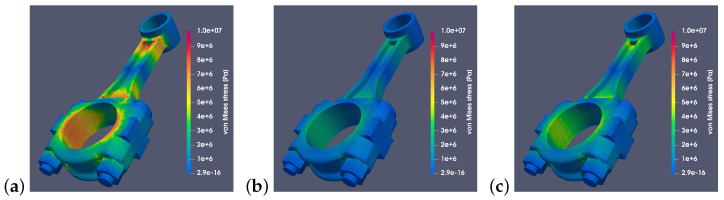
Von Mises stresses for a Suzuki GS650 connecting rod made of (**a**) steel 42CrMo4, (**b**) aluminum alloy 2618, and (**c**) titanium alloy Ti6Al4V with a common stress scale (for angle α=0°).

The results obtained using the FEM are presented in Figure 9, Figure 10, Figure 11, Figure 12 and Figure 13. Figure 9 shows the stress distribution $\sigma_{v}$ for the RÁBA-MAN D2356 HM6U and Suzuki GS650 connecting rods. The analysis was performed assuming that both connecting rods were made of steel 42CrMo4, the material properties of which are specified in Table 2. Figure 9a shows the RÁBA-MAN connecting rod for the angle α=0°, while Figure 9b shows the Suzuki connecting rod for the angle α=90°. The calculations showed that the maximum stresses in the Suzuki connecting rod were approximately three times higher than those in the RÁBA-MAN connecting rod. This is due to the fact that, despite its smaller size, the Suzuki connecting rod operates at a rotational speed 4.5 times higher, and inertia acceleration is proportional to the product of the radius and the square of the angular velocity. It is evident that, in this specific case, the smaller dimensions do not compensate for the increase in angular velocity, resulting in significantly higher stress values.

Figure 10 and Figure 11 show the stresses occurring in both connecting rods for different values of the angle α and assuming that the connecting rods were made of steel 42CrMo4. The image sequences show how the stress system changes during the rotation of the crankshaft by a given angle.

Figure 12 and Figure 13 show stresses when different materials were used. Connecting rods made of steel 42CrMo4, aluminum alloy 2618, and titanium alloy Ti6Al4V were analyzed. For each connecting rod model, an identical scale was used to show how significantly the value of stresses generated by the inertia force differs depending on the material used. The presented simulation results clearly show that a significant portion of the critical stresses of Suzuki connecting rod was localized on the surface of the large hole. The magnitude and nature of these stresses affected the deformation of the hole and the bearing located within it. This, in turn, may negatively impact the tribological processes occurring during the operation of the crank–piston system. However, this aspect goes beyond the scope of this article and may be a subject of further research.

It should be noted that in the presented simulations, for the demonstration purposes, connecting rods were used, structurally equipped with removable connecting-rod foot-bearing covers but defined as a single homogeneous rigid body. The cover’s mounting screws represented only the appropriate mass, and the connecting rods behaved like a rigid body. The connecting rods of a homogeneous rigid body structure are usually used when rolling bearings are used on the connecting rod’s foot. There are, of course, no obstacles to testing split connecting rods composed of several elements, taking into account the loads acting between these elements.

## 5. Discussion

The model proposed in this article was designed with three main applications in mind. The first was a thorough analysis of the dynamics of the crank–piston system. For this purpose, direct formulas for the value of the inertia force that appear due to the movement of the connecting rod were derived.

The second application was the modeling of stresses generated in the connecting rod by the inertia force. The derived formulas allowed us to determine the vector field of body force:

(58) $$\begin{array}{c} f\left(s_{1},s_{2},s_{3}\right)=\rho R\omega^{2}\left[\begin{array}{c} c^{\prime \prime }\left(\alpha \right)-\frac{s_{1}}{L}c^{\prime \prime }\left(\alpha \right)+\frac{s_{2}}{L}\sin\alpha +\cos\alpha \\ -\frac{s_{2}}{L}c^{\prime \prime }\left(\alpha \right)-\frac{s_{1}}{L}\sin\alpha \\ 0 \end{array}\right], \end{array}$$

where the $s_{1}$ axis passes through the centers of the small end (point A) and the big end (point B) of the connecting rod. The $s_{3}$ axis coincides with the rotation axis of the small end, and the $s_{2}$ axis is perpendicular to the $s_{1}$ and $s_{3}$ axes and passes through the center of the small end (point A). In this case, using the system of Equations (48)–(50), it becomes possible to determine the stresses, strains, and displacements of the connecting rod resulting from the action of inertial force.

The third important issue was the influence of the inertia force on the generation of the torsional vibrations of the crankshaft. The moment of force responsible for causing these vibrations is a vector product of the radius vector $\vec{OB}$ of length *R* and the inertia force given by Equation (46). Since

(59) $$\begin{array}{c} \vec{OB}=R\left[\begin{array}{c} -\cos\alpha \\ \sin\alpha \\ 0 \end{array}\right], \end{array}$$

the moment of force generating the torsional vibrations of the crankshaft can be expressed using the following formula

(60) $$\begin{array}{c} M=R^{2}\omega^{2}\left(\left(m_{c}-\frac{1}{L}\;m_{c,1}\right)\left[\begin{array}{c} 0\\ 0\\ -\sin\alpha (\cos\alpha +c^{\prime \prime }\left(\alpha \right)) \end{array}\right]\right.\\ -\left.\frac{1}{L}\;m_{c,2}\left[\begin{array}{c} 0\\ 0\\ 1 \end{array}\right]+\frac{1}{L}\;m_{c,2}\left[\begin{array}{c} 0\\ 0\\ \cos\alpha (\cos\alpha +c^{\prime \prime }\left(\alpha \right)) \end{array}\right]\right). \end{array}$$

Since the components *x* and *y* of the moment of inertia vector are zero, its value can be unequivocally represented as a scalar

(61) $$\begin{array}{c} M_{z}=R^{2}\omega^{2}\left(-\left(m_{c}-\frac{1}{L}\;m_{c,1}\right)\sin\alpha \left(\cos\alpha +c^{\prime \prime }\left(\alpha \right)\right)\right.\\ -\left.\frac{1}{L}\;m_{c,2}\left(1-\cos\alpha (\cos\alpha +c^{\prime \prime }\left(\alpha \right))\right)\right). \end{array}$$

It should be noted that, so far, the authors have not conducted real experiments to verify the accuracy of the proposed model. This is because constructing a specialized test setup is necessary for this purpose, and its design must be adapted to the process of collecting stress data from an operating connecting rod. Experimental verification is certainly one of the possible directions for further research in this field.

## 6. Conclusions

The article presented a mathematical model of the inertia force acting on the connecting rod during its movement. The derived dependencies assumed that the shaft’s rotational speed with which the connecting rod rotated was constant in time. The main area of application of the developed model is the measurement and analysis of the stresses occurring in the connecting rod during engine operation and the vibrations generated by the inertia force of the shaft. Both mentioned issues are closely related to the reliability of crank–piston systems and the tribological processes that occur within them. This is particularly important in the era of growing design requirements, for which one of the priorities is to slim down the structure while increasing performance.

The numerical analysis conducted for sample connecting rods and their operating parameters clearly indicated that even a small connecting rod weighing 300 g could generate inertia forces of up to 10 kN. The primary reason for this is that these forces are proportional only to the first power of the crank radius dimension and the square of the rotational speed.

Surprisingly, a nearly 20-time heavier connecting rod, rotating at 1000 rpm, generated only about twice the inertia forces. The developed FEA model further demonstrated that connecting rods made of high-density materials and operating at high rotational speeds were more susceptible to the negative effects of inertia-induced stresses.

The simulations revealed that the small Suzuki connecting rod experienced nearly four-time higher stresses due to inertia forces than the large RÁBA-MAN diesel engine connecting rod. Of course, inertia forces are not as large as the gas forces generated by the combustion of the fuel–air mixture. However, it should be noted that inertia forces act continuously with cyclic directional changes, whereas gas forces are largely limited to the direction aligned with the longitudinal axis of the connecting rod.

The authors’ work resulted in a tool useful in designing the crank–piston components. It allows for a very precise analysis of the correctness of the connecting rod design and its optimization. This is of particular importance, considering the efforts to increase the engine efficiency and their environmental friendliness. The proposed tool has the potential to analyze complicated cases of connecting rods with an even more complex structure and kinematics of movement. An example of this is the increasingly popular connecting rods equipped with a system for changing the position of the piston’s pin hole axis, which allows one to change the engine compression ratio.

It should be noted that the introduced computational model assumed that the connecting rod operated in a system with a constant rotational speed. All the performed differentiations were based on the assumption that the function ω did not change over time. This represents a fundamental limitation of the proposed model, which will not accurately reflect the actual inertia forces and the resulting stresses in cases where the system’s rotational speed varies. Therefore, developing a model that accounts for changes in angular velocity over time should be the next step in refining the presented approach.

## Figures and Tables

**Table 1:** Parameters of engines and their connecting rods.

	RÁBA-MAN D2356 HM6U	Suzuki GS650
Displacement [cm^3^]	10,690	650
Maximum power [kW]	160.6	53.3
Cylinder diameter [mm]	121	62
Shaft’s rotational speed [rpm]	2100	9500
ω [rad/s]	70π	316.7π
*R* [mm]	75	27.9
*L* [mm]	275	100
λ [-]	0.2727	0.2790
$m_{c}$ [g]	5383.84	310.0

**Table 2:** Material parameters used in the numerical simulations.

	42CrMo4	Aluminum 2618	Ti6Al4V
Young’s modulus [Pa]	210 × $10^{9}$	74 × $10^{9}$	114 × $10^{9}$
Poisson’s ratio	0.29	0.33	0.34
Density [kg·m^−3^]	7800	2760	4430

**Table 3:** Mesh size independence analysis in the simulation calculation of the connecting rod.

RÁBA-MAN D2356 HM6U		Suzuki GS650	
**Mesh Size [mm]**	**Maximum** $\sigma_{v}$ **[MPa]**	**Mesh Size [mm]**	**Maximum** $\sigma_{v}$ **[MPa]**
1.20	5.634	0.360	15.063
1.40	5.494	0.420	15.007
1.70	5.203	0.540	13.094
2.00	4.347	0.720	12.469
2.50	3.874	0.900	12.666

## Data Availability

The data presented in this study are openly available in https://github.com/achmie/conrod (accessed on 1 March 2025).

## Appendix A

Below is a fragment of Python code that reads the three-dimensional mesh of the connecting rod from an MSH file saved in the GMSH version 2.2 ASCII format.
  # Load the~mesh from STL file
  mesh = meshio.read("conrod_suzuki_si.msh")
  #mesh = meshio.read("conrod_rabaman_si.msh")

  # Extract points and cells (tetrahedrons)
  points = np.array(mesh.points)
  cells = mesh.cells_dict["tetra"]
		

After loading the tetrahedral mesh, the total volume of the connecting rod *V*, its mass $m_{c}$, and the moments $m_{c,1}$ and $m_{c,2}$ are determined based on the model geometry and material density. The following code fragment performs these calculations.

# Numerical integration - summation by tetrahedrons
  for i in range(len(cells)):
    # Take tetrahedron vertices indices
    ti = cells[i]
    # Convert vertices indices to (x,y,z) coordinates:
    pi = points[ti, :].T
    # Convert vertices A,B,C,D to 3 vectors with origin at A
    wi = pi[:, 1:4] - pi[:, 0:1]
    # Volume of tetrahedron ABCD as 1/6 of vectors volume
    Vi = abs(np.linalg.det(wi)) / 6
    V += Vi
    # Compute tetrahedron mass
    mci = rho * Vi
    mc += mci
    # Tetrahedron ABCD centroid
    d = np.sum(pi, axis=1) / 4
    # Integration by s1*dm and s2*dm
    mc1 += d[0] * mci
    mc2 += d[1] * mci

## Appendix B

The code implementing the problem (56) in FreeFem++ can be written as shown below.

 // Macro, epsilon(ux,uy,uz)’ * epsilon(vx,vy,vz) = \
  epsilon(u) : epsilon(v)
macro epsilon(ux,uy,uz) [ dx(ux), (dy(ux)+dx(uy))/2, \
  (dz(ux)+dx(uz))/2, (dx(uy)+dy(ux))/2, dy(uy), \
  (dz(uy)+dy(uz))/2, (dx(uz)+dz(ux))/2, (dy(uz)+dz(uy))/2, \
  dz(uz) ] //

// Macro, div(ux,uy,uz) = \nabla \cdot [ux,uy,uz]
macro div(ux,uy,uz) ( dx(ux) + dy(uy) + dz(uz) ) //

// ...

// Body force (density * acceleration)
func fx = rho*omega*omega*R*(l*cos(2*alpha) -
  (1/L)*x*l*cos(2*alpha) + (1/L)*y*sin(alpha) + cos(alpha));
func fy = rho*omega*omega*R*(-(1/L)*y*l*cos(2*alpha) -
  (1/L)*x*sin(alpha));
func fz = 0;

// Equilibrium state (weak formulation)
solve ConRod3D([ux,uy,uz], [vx,vy,vz])
  = int3d(Th)(
    lambda * div(ux, uy, uz) * div(vx, vy, vz)
    + 2.*mu * ( epsilon(ux, uy, uz)’ * epsilon(vx, vy, vz) )
  )
  - int3d(Th)(
    [fx,fy,fz]’ * [vx,vy,vz]
  )
  + on(212, ux=0, uy=0, uz=0)
  + on(213, ux=0, uy=0, uz=0);
		
